# Functional Thyroid Follicular Cells Differentiation from Human-Induced Pluripotent Stem Cells in Suspension Culture

**DOI:** 10.3389/fendo.2017.00103

**Published:** 2017-05-22

**Authors:** Ayumi Arauchi, Katsuhisa Matsuura, Tatsuya Shimizu, Teruo Okano

**Affiliations:** ^1^Institute of Advanced Biomedical Engineering and Science, Tokyo Women’s Medical University, Tokyo, Japan; ^2^Department of Cardiology, Tokyo Women’s Medical University, Tokyo, Japan

**Keywords:** induced pluripotent stem cells, differentiation, 3D-cell culture, thyroid transcription factor, thyroid follicular cells

## Abstract

The replacement of regenerated thyroid follicular cells (TFCs) is a promising therapeutic strategy for patients with hypothyroidism. Here, we have succeeded in inducing functional TFCs from human-induced pluripotent stem cells (iPSCs) in scalable suspension culture. Differentiation of iPSCs with Activin A treatment produced Sox17- and FoxA2-expressing definitive endodermal cells that also expressed thyroid transcription factors Pax8 and Nkx2-1. Further treatment with thyroid-stimulating hormone (TSH) induced TFCs expressing various types of thyroid proteins including TSH receptor, sodium–iodide symporter, thyroglobulin, and thyroid peroxidase. Interestingly, differentiated cells secreted free thyroxine *in vitro*. These results indicate successful differentiation of human iPSCs to functional TFCs that may enable us to fabricate thyroid tissues for regenerative medicine and disease models.

## Introduction

Although thyroidectomy is widely applied for patients with various types of thyroid diseases, such as thyroid nodular diseases, goiters, and Basedow’s disease, subsequent hypothyroidism remains a highly undesirable problem (1). Currently, patients with thyroid hormone shortage subsequent to thyroidectomy require thyroid hormone replacement therapy *via* oral administration for life. However, as doses are often adjusted according to endogenous hormone levels, patients are not always maintained in euthyroid condition. Therefore, treatment strategies to physiologically supplement thyroid hormone levels are ideal, with thyroid replacement using regenerated tissue expected to be an alternative and radical treatment.

Thyroid tissue is constructed from two types of cells, thyroid follicular cells (TFCs) and parafollicular cells (C-cells) (2). TFCs primarily regulate thyroid functions such as production and secretion of thyroid hormones, including T_3_ and T_4_. A monolayer of TFCs organizes follicles into honeycomb-like structures that produce thyroglobulin (TG), which is modified with iodine into thyroid hormone (2). We previously demonstrated fabrication of the thyroid gland *in vivo* using cell sheet tissue engineering of rat thyroid cells (3–5). Fabricated thyroid cell sheets showed follicle structures on the temperature-responsive culture dishes *in vitro*. Moreover, when transplanted subcutaneously into a rat total thyroidectomy model, regenerated tissues with structures resembling the thyroid gland’s spherical follicular cell layer (with collide inside) and parafollicular cells were observed. Further, serum concentration of free triiodothyronine (T_3_) and thyroxine (T_4_) recovered to levels similar to that of littermates. These findings indicate the integration of suitable cell sources and appropriate tissue engineering technologies potentiates fabrication of functional thyroid tissues to replace thyroid hormone (5).

While it is ideal to transplant a patient’s own thyroid cells after isolating and expanding these cells from tissues resected during surgery, transplantation of over 100 million TFCs is calculated from previous reports in animals (5) to be necessary to supplement reduced thyroid hormone levels observed with hypothyroidism. Furthermore, this strategy might not be suitable for patients with malignant tumors owing to the recurrent risk. Recent advancement of reprogramming technologies has enabled generation of patient-derived induced pluripotent stem cells (iPSCs) that may overcome cell source issues in terms of both quality and quantity (6, 7). Additionally, the development of three-dimensional (3-D) suspension culture strategies facilitates generation of large amounts of differentiated cells, such as iPSC-derived cardiomyocytes and pancreatic b cells, within a single production run. Recently, we developed a scalable 3-D suspension culture system for human iPSCs and succeeded in producing over 100 million human cardiomyocytes, which enabled fabrication of functional cardiac tissues *in vitro* and *in vivo* (8, 9). However, it remains unclear whether human iPSCs can be differentiated into functional TFCs using scalable suspension culture methods.

Members of the thyroid transcription factor (TTF) family include paired box protein 8 (Pax8) and homeobox protein (Nkx2-1, also known as TTF-1) (10), which are known to be essential for thyroid gland development and morphogenesis (11–13). Complete absence of thyroid follicles has been reported in mice lacking *Pax8* or *Nkx2-1* genes (11–13). Meanwhile, overexpression of Pax8 and Nkx2-1 in mouse and human embryonic stem cells (ESCs) or PSCs is reportedly enough to drive TFCs differentiation into thyroid hormone-secreting cells that recover function of hypothyroidism models *in vivo* (14–16). Antonica et al. succeeded in generation of functional TFCs from the Pax8 and Nkx2-1 co-expressed mouse ESCs with Matrigel-supported 3D culture stimulated by thyroid-stimulating hormone (TSH) (14), and also Ma et al. reported the differentiation both of mouse and human PSCs into the functional follicle structural TFCs by Activin A and TSH stimulation (15, 16). Furthermore, as these transcription factors directly regulate expression of thyroid-specific genes including *TG, thyroid-stimulating hormone receptor (TSHR), thyroid peroxidase (TPO)*, and sodium/iodide symporter (*NIS*) (17), specific expression levels of Pax8 and Nkx2-1 might be sufficient for functional TFC differentiation. However, it remains unclear whether these defined factors can induce sufficient upregulation of Pax8 and Nkx2-1 expression to further TFC differentiation.

This study demonstrated direct differentiation of human iPSCs into functional TFCs using defined factors in a scalable suspension culture method. Treatment with Activin A and TSH induced Pax8- and Nkx2-1-expressing cells in cell aggregates through a definitive endoderm intermediate. Furthermore, *PAX8*- and *NKX2-1*-expressing cells also expressed *TG, TSHR, TPO*, and *NIS* and secreted free T_4_ *in vitro*.

## Materials and Methods

### Growth and Maintenance of Human iPSCs

Human iPSCs (253G1) were purchased from RIKEN. These cells were generated from human skin fibroblast by retroviral transduction of *Oct3/4, Sox2, Klf4* (7), and maintained on a feeder-cell layer of mitomycin C-treated mouse embryonic fibroblasts (ReproCELL). Subculture passages were performed every 2 days at a 1:3 ratio in Primate ES Medium (ReproCELL) supplemented with 5 ng/ml basic fibroblast growth factor (ReproCELL). The culture medium was changed every day. Undifferentiated state was assessed routinely and before starting differentiation experiments by qPCR for *Nanog, Oct-4* gene expression.

### Differentiation of Human iPSCs into Definitive Endoderm and TFCs

First, we collected human iPSCs using a dissociation solution (ReproCELL) and suspended these cells into small aggregations. To aggregate, cells were harvested in mTeSR1 (Stemcell Technologies) with 10 µM Y-27632 (Wako) and then stirred in a 30-ml volume-standardized bioreactor (ABLE Co.) for 2 days to form embryoid bodies (EBs). These EBs were then cultured for 3 days to induce definitive endoderm in StemPro-34 Media (Gibco) supplemented with StemPRO-34 Nutrient Supplement (Gibco), 400 µM monothioglycerol (Sigma-Aldrich), 2 mM l-Glutamine (Gibco), 50 μg/ml l-Ascorbic acid (Sigma-Aldrich), 100 ng/ml Activin A (R&D systems), and on the first day only, 3 µM CHIR99021 (Stemcell Technologies). Later, differentiation of EBs into TFCs was performed in endodermal medium without Activin A and with addition of the following nutrients: 1 mU/ml bovine thyrotropic hormone (TSH) (Sigma-Aldrich), 50 ng/ml recombinant human IGF-1 (Invitrogen), 10 µg/ml human recombinant insulin (Gibco), 6 µg/ml transferrin (Roche Applied Science) and 10^−8^ M hydrocortisone (Calbiochem). Samples were collected on culture days 0, 5, 10, 15, 20, and 25. The medium was changed every other day.

### RNA Extraction and Real-time Reverse-Transcription Polymerase Chain Reaction (RT-PCR)

To extract total RNA from EBs, an RNeasy^®^ Plus Mini Kit (Qiagen) was used according to the manufacturer’s protocol. cDNA synthesis was completed using a First-Strand cDNA Synthesis Kit (OriGene). Primer pairs and TaqMan MGB probes were designed for human *SOX17* (Hs00751752_s1), *FOXA2* (Hs00232764_m1), *PAX8* (Hs01015257_g1), *NKX2-1* (Ttf-1) (Hs00968940_m1), *SLC5A5* (*Nis*) (Hs00166567_m1), *TPO* (Hs00892519_m1), *TG* (Hs00174974_m1), *TSHR* (Hs01053846_m1), and *GAPDH* (Hs00751752_s1) using a TaqMan gene expression assay (Applied Biosystems). RT-PCR was performed with SYBR^®^ Green qPCR Master Mix (Applied Biosystems) and a StepOnePlus™ Real-time PCR System (Applied Biosystems). mRNA expression levels were analyzed in real time using the 2^−ΔΔCT^ method normalized to *GAPDH* expression as an internal control. Results were confirmed in three different samples (*n* = 3) for each time-dependent collection and all samples were tested in duplicate.

### Flow Cytometry

Collected EBs were washed with PBS (Sigma-Aldrich) and treated with 0.25% Trypsin-EDTA (Thermo Fisher Scientific) for 10 min at 37°C to disperse EBs into single cells. Following 2% paraformaldehyde (PFA) fixation for 10 min, single cells were fixed with 4% PFA for 20 min and then harvested as a cell pellet by centrifugation at 430 × *g* for 3 min. Cells were incubated in blocking solution, consisting of 5% donkey serum and 0.1% Triton™-X (Sigma-Aldrich) in PBS, for 30 min at room temperature and then divided into several tubes for staining. Cells were then incubated with primary antibodies at 4°C overnight. For our double-immunostaining procedure, goat anti-human Sox17 (AF1924 R&D systems, 1:50) and rabbit anti-human FOXA2 conjugated to Alexa Fluor^®^ 647 (ab193879 Abcam, 1:200), or goat anti-human Pax8 (ab13611 Abcam, 1:200) and rabbit anti-human TTF-1 (ab76013 Abcam, 1:200) were used. For negative controls, normal goat IgG (sc2028 Santa Cruz Biotechnology), normal rabbit IgG (sc2027 Santa Cruz Biotechnology), and normal rabbit IgG conjugated to Alexa Fluor 647 (sc24647 Santa Cruz Biotechnology) were applied.

Finally, following three rinses with blocking solution, cells were incubated for 60 min at room temperature with secondary antibodies, including anti-goat Alexa 488 (705546147 Jackson ImmunoResearch, 1:200), anti-rabbit Alexa 594 (711586152 Jackson ImmunoResearch, 1:200), and Hoechst 33342 (H3570 Thermo Fisher Scientific, 1:500) nuclei stain. Prepared samples were evaluated using a Gallios (Beckman Coulter) and obtained results were analyzed by Kaluza (Beckman Coulter).

### Immunostaining

Embryoid bodies were washed with PBS and fixed with 4% PFA for 20 min. Cells were incubated for 60 min at room temperature with blocking solution, consisting of 5% donkey serum and 0.1% Triton-X in PBS, and divided into 1.5-ml microtubes for staining. EBs were incubated with primary antibodies at 4°C overnight and then rinsed three times with blocking solution before applying secondary antibodies for 60 min at room temperature. Primary antibodies against Sox17, FoxA2, Pax8, and TTF-1, as well as negative control and secondary antibodies, employed the same materials and dilution as used for flow cytometry. To identify thyrocytes, thyroid-specific antibodies including mouse anti-human TSHR (ab6044 Abcam, 1:50), mouse anti-human NIS (ab17795 Abcam, 1:50), rabbit anti-human TG (ab156008 Abcam, 1:50), and normal mouse IgG (sc2025 Santa Cruz Biotechnology) was applied for the negative controls. EBs were washed with PBS, spread on the surface of a glass slide, and then mounted with GelMount™ (Biomeda). Finally, all of the immunostained samples were examined under confocal microscope (Olympus).

### Enzyme-Linked Immunoassay (ELISA)

Medium was sampled at the end point of human iPSC differentiation. To measure the level of human-free thyroxine (free T_4_) in medium, a Human-Free Thyroxine ELISA Kit (MyBioSource) was employed according to the manufacturer’s protocol. Results were confirmed in three different samples (*n* = 3) for each collected medium and without cell-cultured medium. All samples were tested in duplicate.

### Statistical Analysis

Values are expressed as mean ± SD. Data were plotted using Microsoft Excel, and statistical analyses were performed using ANOVA, *post hoc* Tukey’s test and unpaired Student’s *t*-test. Values of *p* < 0.05 were considered statistically significant.

## Results

### Differentiation of Human iPSCs into Definitive Endoderm

**Figure 1:**
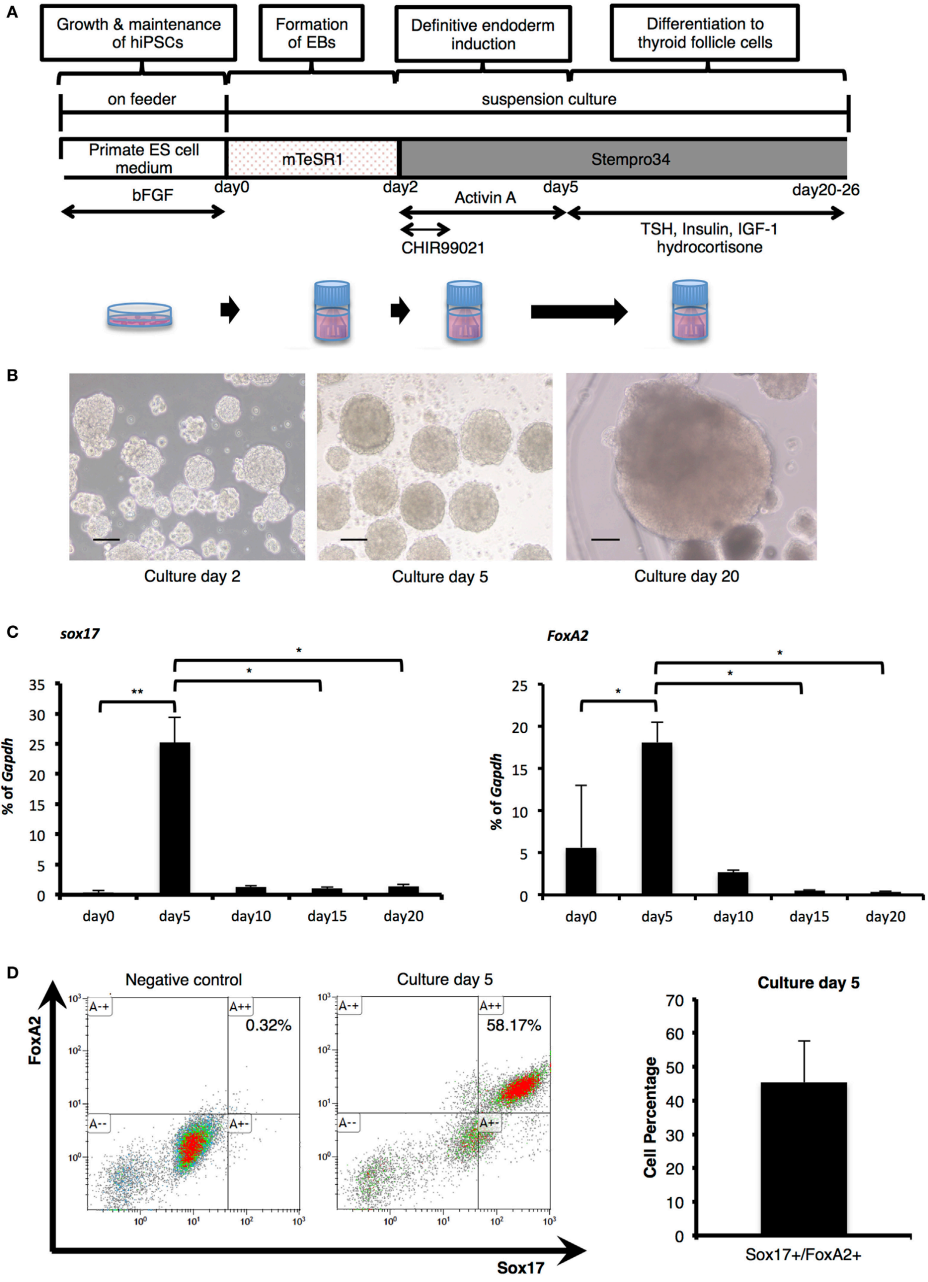

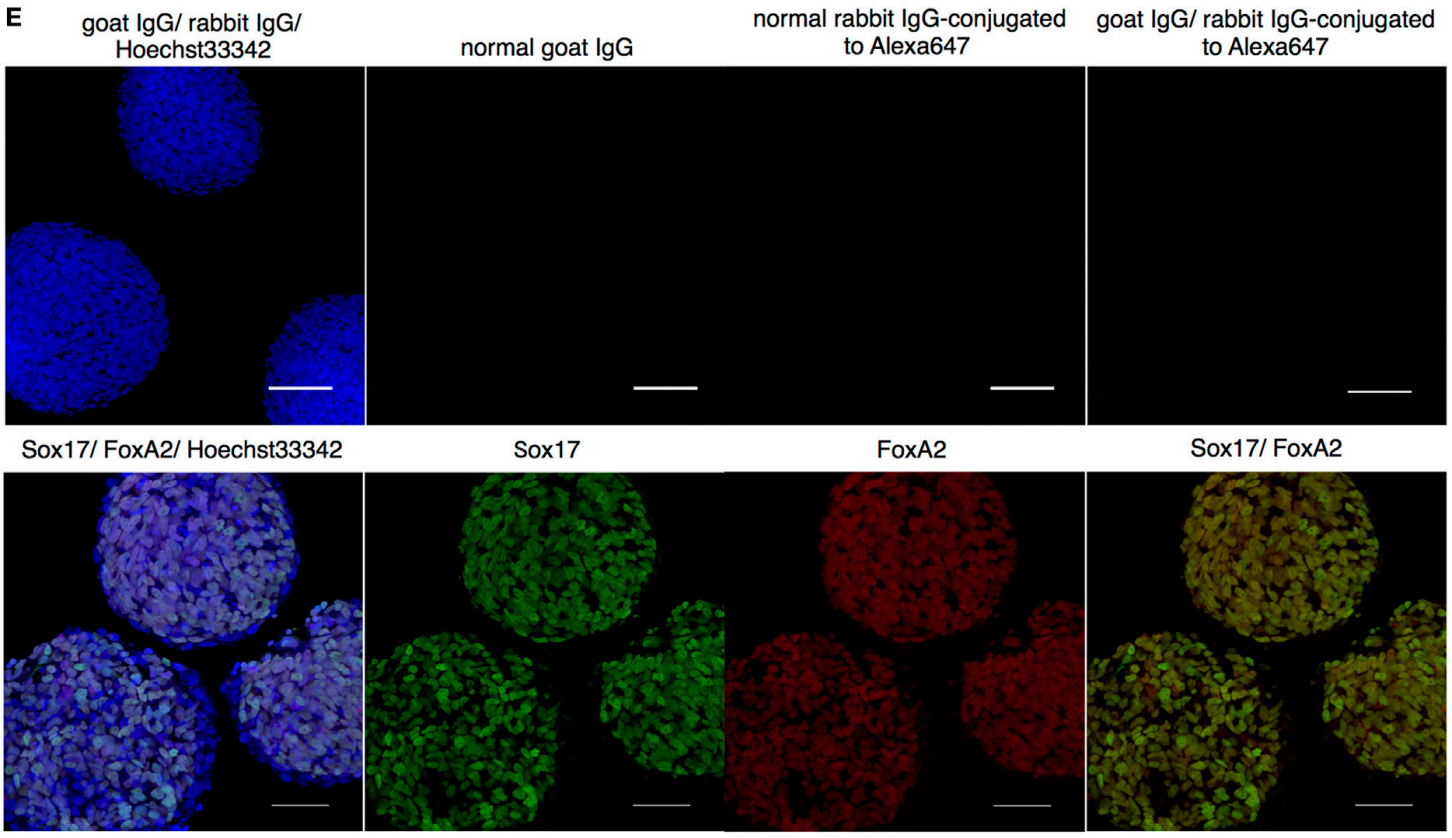
**Endodermal differentiation of human induced pluripotent stem cells (iPSCs)**. **(A)** Schematic diagram of the induction protocol for definitive endoderm production from human iPSCs. **(B)** Embryoid bodies (EBs) collected pre- and post-Activin A exposure, as observed by light microscopy on culture days 2, 5, and 20. Scale bar = 100 µm. **(C)** Real-time reverse-transcription polymerase chain reaction analysis of definitive endodermal markers. Significant differences in *Sox17* were detected between EBs after Activin A exposure (culture day 5) and samples on culture day 0, day 15, and day 20. In addition, *FoxA2* expression on day 5 was significantly increased in comparison with cultures on day 0, and decreased with significant differences between those of day 15 and day 20. Bars indicate average percentage of *Gapdh* gene expression ±SD; (*p < 0.05, **p < 0.01 versus day 5, *n* = 3). Statistical analysis was performed by one-way ANOVA and *post hoc* Tukey’s test. **(D)** Percentage of Sox17- and FoxA2-expressing cells as counted by flow cytometry. After stimulation with Activin A for 3 days, the rate of Sox17 and FoxA2 double-positive cells increased to more than 45%, with a maximum of 58%. Bars indicate average percentage of Sox17- and FoxA2-expressing cells ± SD (*n* = 3). **(E)** Immunostaining of EBs on day 5 with anti-Sox17 and anti-FoxA2 antibodies. Images in the upper line present negative control. Images in the lower show Hoechst33342 for nuclei (blue) and Sox17 (green) and FoxA2 (red). Scale bar = 100 µm in the upper line and 50 µm in the lower.

As thyroid development is known to progress *via* definitive endoderm (18–20), human iPSCs were cultured in medium supplemented with Activin A for endodermal differentiation following formation of cell aggregates in a stirred vessel (Figure 1A). After 2 days in undifferentiated culture conditions, spherical agglomerations increased in size to approximately 100 µm (Figure 1B). The size of these (EBs) increased to more than 200 µm in diameter (Figure 1B) after 5 days (day 3 of differentiation). To confirm endodermal differentiation, expression of *Sox17* and *FoxA2* mRNA was evaluated with RT-PCR analysis. Compared with before differentiation (day 0), both Sox17 and FoxA2 expression significantly increased on day 5, and thereafter decreased until day 20 (Figure 1C). Flow cytometric analysis revealed that more than 45% of cells expressed both *Sox17* and *FoxA2* on day 5 (Figure 1D) and these proteins were co-expressed in nuclei within cell aggregates (Figure 1E). Accordingly, these results indicate successful definitive endodermal differentiation.

### Differentiation of TFCs

**Figure 2:**
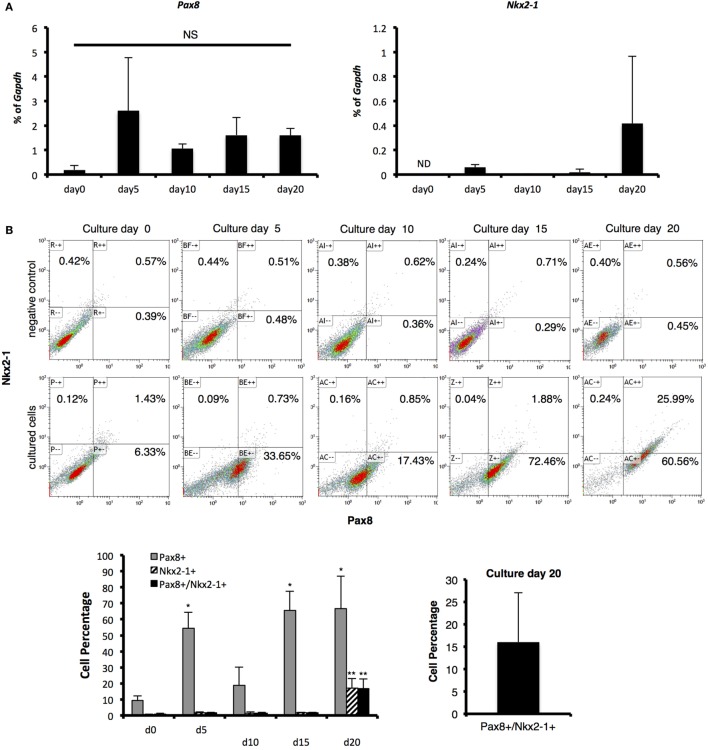

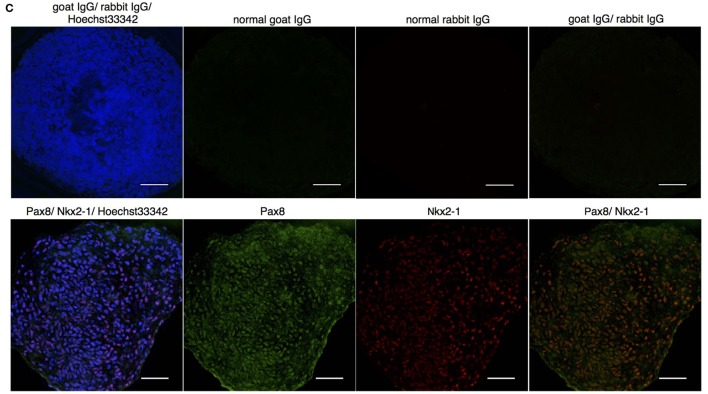
**Identification of thyroid transcription markers**. **(A)** *Pax8* and *Nkx2-1* mRNA expression as measured with real-time reverse-transcription polymerase chain reaction. Levels of gene expression were normalized to *Gapdh*. Bars indicate average percentage of *Gapdh* gene expression ± SD (*n* = 3). ND indicates mRNA expression was not detected and NS indicates not significant. There was no significant difference between all samples in Pax8 by ANOVA. **(B)** Percentage of Pax8 and Nkx2-1 co-expressing cells as counted by flow cytometry. Pax8-positive cells increased in 5 days to more than 50%, which was maintained until day 20, except for a transient decrease on day 10. Percentage of Pax8 and Nkx2-1 double-positive cells increased in a time-dependent manner, resulting in 16% approximately on culture day 20. Negative controls were applied for normal IgG instead of primary antibodies. Bars indicate average percentage of Pax8- and Nkx2-1-coexpressing cells ± SD (*n* = 3). Statistical analysis was performed by one-way ANOVA. **(C)** Immunohistological analysis of Pax8 and Nkx2-1. Images in the upper line present negative controls. Images in lower show Pax8 (green), Nkx2-1 (red), and Hoechst33342 (blue). Scale bar = 50 µm.

**Figure 3:**
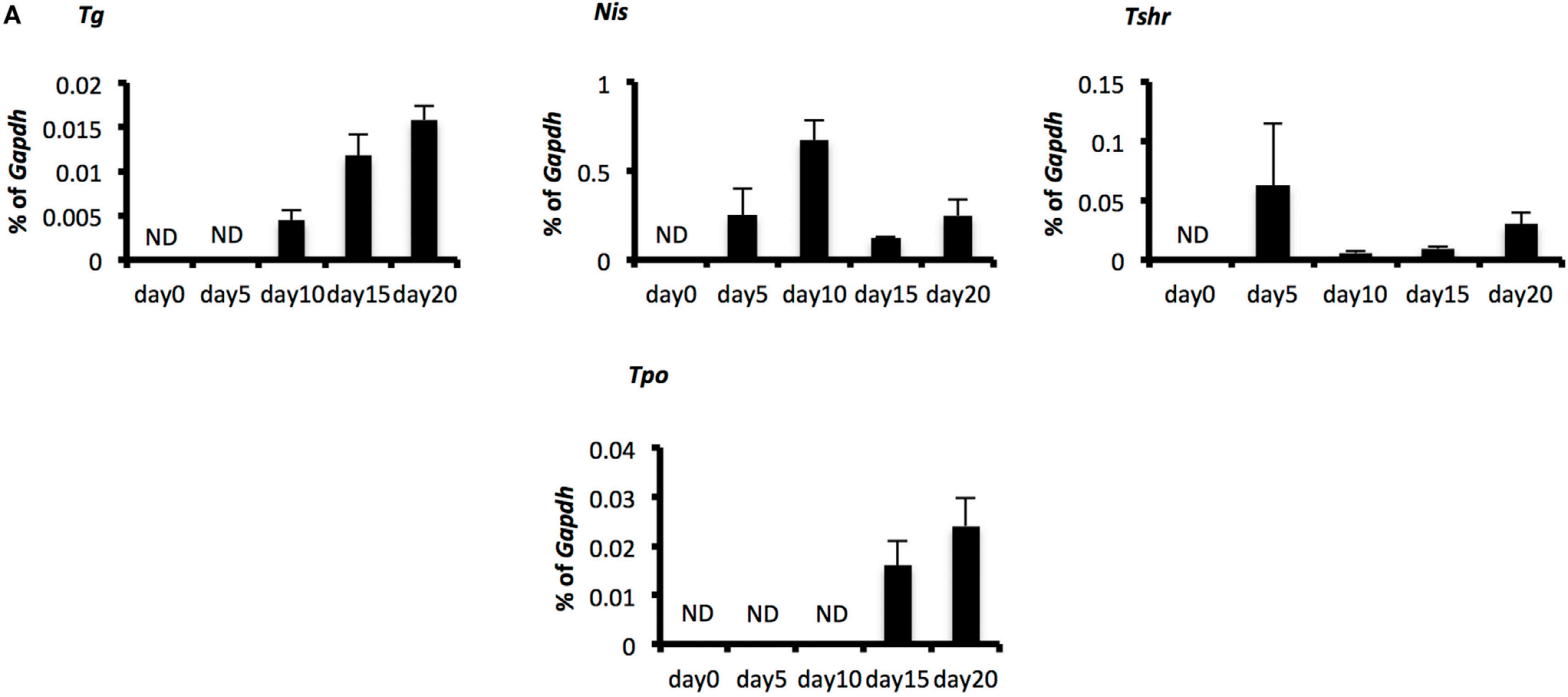

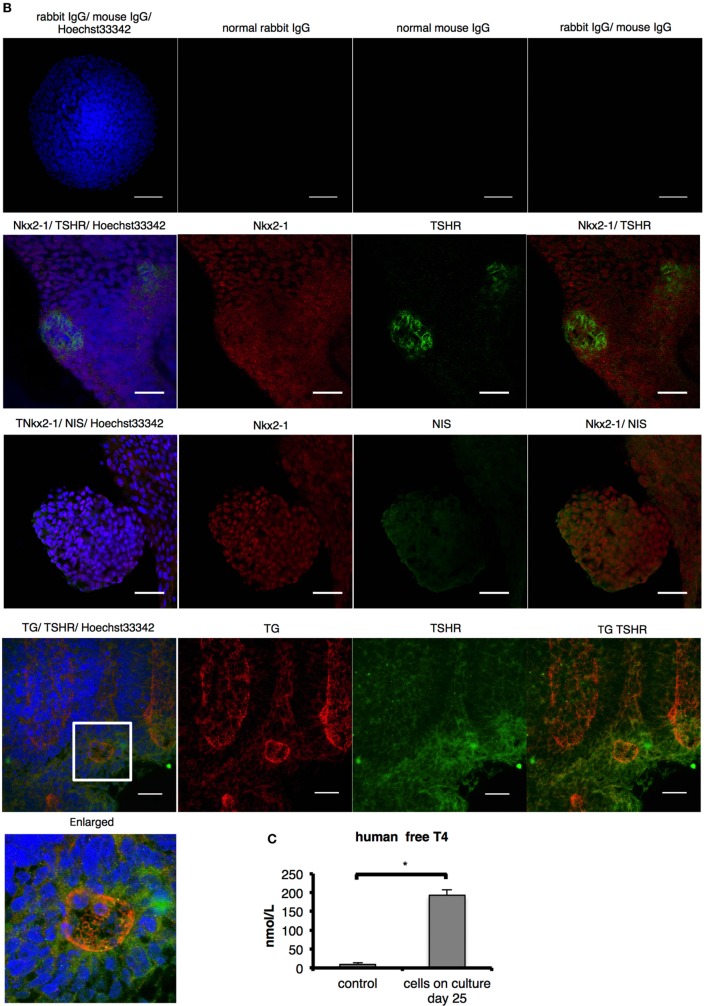
**Evaluation of thyroid specificity with characteristic thyroid markers on culture day 20 and measurement of free T_4_**. **(A)** Levels of thyroid-specific mRNA expression. Bars indicate average percentage of *Gapdh* gene expression ± SD (*n* = 3). ND, not-detected. **(B)** Immunostaining of embryoid bodies on day 20 with thyroid-specific markers NIS, thyroglobulin, and thyroid-stimulating hormone receptor. Images in the top line present negative controls. Scale bar = 50 µm in the top line, 30 µm in the lower lines. **(C)** Levels of human-free T_4_ were higher significantly in the cultured-cell medium than in the control medium (*p < 0.01). Control medium is thyroid-stimulating hormone-supplemented medium before cells were harvested. Bars indicate average-free T_4_ levels ± SD (*n* = 3). Statistical analysis was performed by unpaired Student’s *t*-test.

Thyroid-stimulating hormone is reportedly critical for thyroid development and as *TSHR* mRNA expression was observed in cells on day 5 (Figures 3A,B), we examined whether cells had the potential to differentiate into TFCs at this time point with TSH treatment (Figure 1A). Pax8 mRNA expression levels and the percentage of Pax8-expressing cells were not different between day5 and day20 (15 days with TSH treatment), suggesting that TSH might not directly affect Pax8 expression. As Pax8 is known to be an essential transcription factor not only for thyroid development but also kidney and urogenital development, we next examined expression of Nkx2-1, another essential transcription factor for thyroid development. *Nkx2-1* mRNA was slightly expressed on day 5 and obvious at day 20, with about 15% of cells positive for Nkx2-1. Although Nkx2-1 is also known to be critical for lung development, almost all Nkx2-1-expressing cells also expressed Pax8 (Figures 2B,C), suggesting that these Pax8 and Nkx2-1 co-expressing cells might be thyroid progenitor cells. These findings suggest continuous treatment with TSH promotes thyroid differentiation in suspension cultures through mainly promote the expression of Nkx2-1.

### Expression of Thyroid-Specific Markers and Secretion of Free T_4_ in Differentiated TFCs *In Vitro*

One report indicates the overexpression of *Pax8* and *Nkx2-1* induces expression of TSHR and NIS in human ESCs even before differentiation and promotes the differentiation to TFCs after TSH treatment (15). As cells on day 20 co-expressed Pax8 and Nkx2-1 mRNA and protein (Figures 2A–C), we next examined whether these cells might possess characteristics of TFCs. Consistent with the previous report examining overexpression of Pax8 (15), *Tshr* and *Nis* were expressed in cells at day 5 to some extent, a feature accompanied by expression of *Pax8* (Figures 2A and 3A). Although expression of *Tg* and *Tpo* was not observed on day 5, it was observed thereafter until day 20 (Figure 3A). Immunostaining of differentiated cells on day 20 revealed expression of NIS surrounding the nuclei of NKX2-1 positive cells, and TSHR protein was expressed in many Nkx2-1-expressing cells (Figure 3B). Moreover, TSHR-positive cells gathered around cells expressing TG inside their cytoplasm (Figure 3B). Secretion of thyroid hormone is the most important function of TFCs. Analysis of culture medium harvested 25 days after differentiation culture by ELISA showed significant increases in human-free T_4_ compared with control medium (differentiation culture medium not used for cultivation; *p* < 0.01, Figure 3C). These findings suggest 3-D suspension culture might successfully induce functional TFCs.

## Discussion

In the present study, we demonstrated the direct differentiation of TFCs from human iPSCs using defined factors in a scalable suspension culture method. Transient treatment with Activin A induced expression of Pax8 and TSHR, whereas subsequent treatment with TSH induced the differentiation of TFCs expressing TG, TPO, TSHR, and NIS. Moreover, differentiated cells secreted free T_4_ *in vitro*.

Thyroid tissues are known to differentiate from definitive endoderm. As reported in a previous study of endoderm differentiation (21), treatment with Activin A and CHIR99021 induced Sox17- and FoxA2-expressing definitive endoderm cells in the present study. Surprisingly, we observed expression of *Pax8* and *Tshr* as early as the definitive endoderm stage (day 5). As several studies have reported promotion of Pax8 and Nkx2-1 expression with Activin A treatment (22, 23), the essential role Activin A directly plays on differentiation of both endoderm and TFCs has been established in our study. Precise mechanisms underlying TSHR expression on day 5 remain unclear. However, as we observed a very small number of *Nkx2-1*-expressing cells on day 5 and this transcription factor is known to bind to the promoter region of TSHR to promote expression (24), activation of Nkx2-1 by Activin A may also drive expression of TSHR on day 5. As thyroid placode cells co-express Pax8 and Nkx2-1 in the pharyngeal floor to be distinguished from other types of cells (25, 26), Pax8 and Nkx2-1 are particularly dealt with indispensable factors for morphogenesis of the thyroid gland (26–28) and estimating thyroid progenitors (29). Therefore, cells co-overexpressing Pax8 and Nkx2-1 have often been applied to study execution of thyroid gland development (15, 16, 30). After treatment with TSH for 15 days (day 20 of culture), we confirmed abundant Pax8 and Nkx2-1 mRNA and protein expression, and about 15% of cells were positive for both Pax8 and Nkx2-1. These results indicate Pax8 and Nkx2-1 are regulated by Activin A stimulation and afterward controlled by the TSH/TSHR pathway. As known as an established fact, Nkx2-1 is expressed in the multiple kinds of tissues and cells, not only thyroid and parathyroid, but the fourth branchial pouch, ultimobranchial body, lung, posterior pituitary, hypothalamus, and trachea (17, 25, 29). Though the current results showed that the percentage of Pax8-negative/Nkx2-1-positive cells was rare, the possibility of the existence of respiratory endoderm or the restricted brain cells cannot be kicked out. As well, Pax8 expresses not only in the developing thyroid gland but kidneys and myelencephalon (17, 26, 27, 31) and plays an essential part in the folliculogenesis of the thyroid gland (12). Therefore, the most of the collected cells, Pax8-positive/Nkx2-1-negative cells might include the developing kidney or neural tissue. At last, Pax8-negative/Nkx2-1-negative cells would include other than endodermal cells.

Expression of other TTFs, such as haematopoietically expressed homeobox (Hhex) and forkhead box E1 (FoxE1) proteins (17, 19), may modulate thyroid-specific genes along with TSH treatment. As expression of FoxE1 is strongly dependent upon Pax8 in thyroid progenitors (13), expression of Pax8 activated by the defined factors used in the present study might be sufficient to regulate transcription of other thyroid-specific genes.

Nkx2-1 is expressed not only in thyroid tissue but also in lung. Recent reports have reiterated the importance of a negative expression correlation between FoxA2 and Nkx2-1 for specification of thyroid tissues (19). Upregulation of Nkx2-1 accompanied by the downregulation of FoxA2 is important for thyroid development, while maintenance of FoxA2 expression in Nkx2-1-expressing cells is essential for lung development (32). In this study, we observed downregulation of FoxA2 after treatment with TSH, which contributed to the efficient differentiation of TFCs.

In the present study, we supplemented TSH in the culture medium to promote differentiation of TFCs from human iPSCs. However, the importance of TSH for thyroid development remains an open question. Some reports indicate subsequent treatment with TSH after transfection of *Pax8* and *Nkx2-1* promotes differentiation of mouse and human ESCs into functional thyroid cells (15, 16, 23, 30, 33–36). More recently, Kurmann et al. reported that TSH treatment promoted the further differentiation of thyroid progenitor cells into functional TFCs in mouse and human ESCs without transfection of TTFs (37). In contrast, Postiglione et al. reported the development of normal-sized thyroid glands in both TSH-deprived and TSHR-knockout mice. However, expression of TPO and NIS was downregulated, suggesting that the TSH/TSHR pathway may be important to regulate TPO and NIS gene expression but is not essential for thyroid development in the mouse embryo (38). In this study, bimodal upregulation of *TSHR* expression was observed at day 5 and day 20. This trend might be enhanced by TTFs such as *Pax8* and *Nkx2-1* in this first 5 days. Meanwhile, subsequent TSH treatment may enhance *TSHR* expression along with upregulation of TTFs, potentially leading to the expression of genes related to thyroid maturation, including *NIS, TPO*, and *TG, via* reciprocal TSH/TSHR pathway activation. In the present study, we successfully observed the secretion of free T_4_ in differentiated TFCs *in vitro*. Though differentiated TFCs did not present typical follicle structures *in vitro*, as cells co-expressing TG and TSHR gathered inside EBs, we examined whether these cells could develop and mature into functional TFCs under folliculogenesis.

The thyroid gland maintains its characteristic honeycomb-like structure by organizing follicles gathered within its lining of follicular epithelial cells (2). Upon a review of the literature, TFCs have primarily been differentiated using traditional adherent culture methods on dishes (33, 34, 36) or embedded in Matrigel^®^ as a 3-D environment (14–16, 32, 37). One of the most unique points in our study of TFC induction is the adoption of 3-D stirred culture methods for all processes. We previously reported the effectiveness of 3-D suspension culture to produce large amounts of cardiomyocytes from human iPSCs, especially for its ease of scalability for cell proliferation (8). To supplement thyroid hormone levels associated with hypothyroidism, it has been estimated that more than 1 × 10^8^–1 × 10^10^ TFCs are required per person (5, 39, 40). Therefore, we anticipate utilizing bioreactor will contribute to regenerative medicine development for thyroid dysfunction by allowing effective production of a large enough number of TFCs. Though issues still remain for cell purification and tumorigenesis, at least with regard to organization of the thyroid gland, we previously succeeded in fabricating rat functional thyroid tissue using cell sheet tissue engineering (5). In the future, our combination of suspension culture and cell sheet technology will facilitate production of human iPSC-derived thyroids capable of rescuing hypothyroidism.

## Author Contributions

AA and KM conceived the study. AA performed the experiments and analyzed the data. AA, KM, TS, and TO wrote the manuscript.

## Conflict of Interest Statement

TO is a founder and director of the board of CellSeed Inc., which licenses technologies and patents from Tokyo Women’s Medical University. TO is also a shareholder of CellSeed Inc., Tokyo Women’s Medical University and is receiving research funds from CellSeed Inc. TO, TS, and KM are inventors of bioreactor systems. Tokyo Women’s Medical University, TO, TS, and KM receive a license fee from ABLE Corporation.
